# Improving Global Outcomes in Cervical Cancer: The Time Has Come for International Federation of Gynecology and Obstetrics Staging to Formally Incorporate Advanced Imaging

**DOI:** 10.1200/JGO.2016.007534

**Published:** 2017-03-21

**Authors:** Shruti Jolly, Shitanshu Uppal, Neerja Bhatla, Carolyn Johnston, Katherine Maturen

**Affiliations:** **Shruti Jolly**, **Shitanshu Uppal**, **Carolyn Johnston**, and **Katherine Maturen**, University of Michigan, Ann Arbor, MI; and **Neerja Bhatla**, All India Institute of Medical Sciences, New Delhi, India.

Worldwide, cervical cancer is the fourth most common cause of cancer death in women. Approximately 85% of all new cervical cancers and 87% of all cervical cancer deaths occur in low- and middle-income countries.^1^ Although cervical cancer is decreasing in the United States and other industrialized countries, the incidence and mortality remain high in many developing countries, as a result of a lack of screening and inadequate treatment services. Global cancer statistics report that the cervical cancer age-standardized mortality rate per 100,000 is 2.5 times higher in less developed areas compared with more developed areas (8.3 *v* 3.3, respectively). Cervical cancer is the second most commonly diagnosed cancer and the third leading cause of cancer death among women in less developed countries.^2^

Recognizing these global disparities, ASCO recently created tiered treatment guidelines specifically for the management and care of women with invasive cervical cancer, stratified by availability of medical resources.^3^ Similar guidelines for screening are also under way. This realistic approach both acknowledges the tremendous variety of levels of care available globally and marshals advanced technologies where available to optimize patient care. This perspective, rather than limiting the higher end of care by structuring it in a one-size-fits-all manner designed for low-resource medical systems, instead articulates and illustrates pathways for growth and development for systems at several levels. These are important steps to minimize global disparities. Yet, to maximize the capacity of medical advances to improve health, detailed and specific outcomes assessment is needed. Outcomes assessment, in turn, requires a robust staging system. We present a commentary on the clinical limitations posed by the current commonly used cervical cancer staging system using International Federation of Gynecology and Obstetrics (FIGO) –staged clinical scenarios and the implications for care of individual patients and broader populations. Finally, we propose a tiered staging modification to indicate the level of imaging used for staging based on the resource setting as defined by ASCO.

## FIGO Staging

Staging serves two important purposes, namely, to guide the management and to assess the prognosis of any cancer. Staging systems should ideally enable comparison of treatment outcomes across countries and continents. Generally, clinical staging of cancer is determined based on the physical examination, imaging tests, and biopsies of the affected area. This allows complete assessment of the location of the primary tumor, including tumor size and extent, lymph node involvement, and evaluation of distant metastatic disease. On the basis of the staging, definitive versus palliative treatment recommendations are made. These recommendations vary widely if the staging system does not account for locoregional and distant disease on the basis of available radiologic evidence.

For cervical cancer, as indeed for all gynecologic cancers, the most widely accepted staging system is the FIGO staging. FIGO includes obstetricians and gynecologists from both developed and developing countries and is a respected and credible voice in the promotion of women’s health around the world. It plays a leading role in improving women’s health in developing countries. The size and influence of the organization allow FIGO to set staging and treatment standards, as well as advocate for improvements in women’s health on a large scale. FIGO was originally founded in 1954, and the cervical cancer stage classification developed shortly thereafter. The staging has undergone multiple modifications since that time,^4^ most recently in 2009.^5^ Recognizing the fact that most cases of cervical cancer occur in developing countries where access to medical technology investigative modalities may be limited, FIGO staging is largely clinical in nature and allows, in addition to pelvic examination, only basic investigations including colposcopy, endocervical curettage, hysteroscopy, cystoscopy, proctoscopy, intravenous urography, and x-rays of lungs and skeleton as needed. Suspected involvement of bladder or rectal mucosa must be confirmed by biopsy. Fine-needle aspiration of palpable nodes or masses is allowed; however, laparoscopic or image-guided biopsies are not allowed for clinical staging. Although computed tomography (CT), magnetic resonance imaging (MRI), and/or positron emission tomography (PET) are not mandatory, FIGO acknowledges that these may provide information on nodal status or systemic spread. However, this information does not affect the clinical FIGO stage.^6^

## Successes of the Current FIGO Staging

As is, the current FIGO staging works well in determining treatment recommendation and prognostic information at the extremes of the clinical spectrum—early low-volume disease and overtly metastatic disease. FIGO cervical cancer stages IA1 and IA2 constitute 24.2% of all cervical cancer diagnoses in the United States based on the National Cancer Institute’s SEER data.^7^ In such clinical scenarios, the risk of parametrial involvement and lymph node and distant metastases is low, and most patients will have good outcomes with surgery alone and not require adjuvant therapy. These patients have cancer that is at or below the spatial resolution of contemporary imaging techniques.

At the other extreme, for patients with overt metastatic disease detected on physical examination or limited radiographs as allowable by FIGO, the staging also directs treatment and prognosis appropriately. To be detectable by plain radiography, metastatic disease must be high volume and relatively advanced, and the nuanced information provided by advanced imaging will not fundamentally change the disease course or treatment. These women will undergo chemotherapy and/or palliative care as indicated. SEER data show 6.8% of patients presenting with widely metastatic disease, but this estimate is, of course, limited to the United States.^7^ In less developed areas with patients presenting with advanced cancer at diagnosis, it would be expected that there are significantly more patients with stage IVB metastatic cancer. For the remaining two thirds of patients with cervical cancer stage IB1 through stage IVA (stage IB/I not otherwise specified [30%], II [16%], III [16%], and IVA [2%] in the SEER setting), FIGO staging in the absence of advanced imaging lacks the detail and depth of information needed to guide treatment and document outcomes in well-resourced treatment settings.

## Difficulties With the Current FIGO Staging

We present two clinical staging scenarios in which imaging information fundamentally changes pathways of care, but these distinctions and their basis are not apparent from the FIGO stage alone.

Stage IB1 disease, consisting of a visible lesion confined to the cervix clinically measured to be less than 4 cm in size, contains a heterogeneous group of patients and illustrates this difficulty. In fact, we suggest that this category, because it is defined in the absence of imaging, falls short of the fundamental goal of staging, which is to direct treatment and meaningfully predict outcomes. By contrast, if imaging data are incorporated formally into preoperative clinical assessment, an important separation between two rather different groups of patients can emerge.

Patients with stage IB1 disease can be treated with radiation alone or surgery.^8^ If surgery is chosen as primary treatment, adjuvant radiation is indicated for some patients with larger tumor size, deep cervical stromal involvement, and tumors with lymphovascular space invasion.^9^ Lymphovascular space invasion cannot be diagnosed by imaging, but MRI has superior performance to clinical examination for the diagnosis of tumor size, particularly for endophytic lesions, and has at least a 94% negative predictive value for demonstrating intact cervical stroma.^10-14^ Thus, accurate preoperative risk stratification using MRI can reduce the number of inappropriate surgeries and subsequent need for multimodality treatment as well as attendant morbidity for these patients. Both MRI and [^18^F]fluorodeoxyglucose ([^18^F]FDG) PET/CT enable prompt noninvasive assessment of lymph nodes.^15,16^ Surgical assessment of nodes is associated with patient morbidity.^17,18^ The intraoperative detection of positive lymph nodes may necessitate patient closure and subsequent referral to radiation therapy. Further, when radiation is selected as primary therapy, the ability to identify cancerous lymph nodes and/or distant disease has major implications for treatment planning as well as being highly prognostic for patient outcome.

A different clinical staging difficulty emerges for patients with FIGO stage IB2 to IVA disease with more locally advanced cervical tumors, for whom definitive chemoradiation with brachytherapy is recommended in high-resource environments. Ultrasound is used as an extension of the physical examination to evaluate tumor size in some practice settings and allows inexpensive, noninvasive detection of hydronephrosis.^19,20^ MRI has superior sensitivity to physical examination both for detecting low-volume parametrial involvement and illustrating the full extent of more advanced disease, including bladder and bowel invasion, which is not always possible to assess on clinical examination alone.^13,21^ By accurately depicting the full extent of disease, MRI may impact radiation treatment planning, enabling the radiation oncologist to evaluate whether intracavitary or interstitial brachytherapy will be required. PET/CT scans can also alter patient treatment significantly in these cases by illustrating both pelvic and extrapelvic disease. Borderline or nonenlarged pelvic and/or para-aortic lymph nodes may exhibit [^18^F]FDG uptake, meaning that they need to be included in radiotherapy planning and given higher doses. This not only affects patient overall survival, but also has important ramifications with regard to toxicity of treatment. If distant metastatic disease is noted, then the patient’s entire treatment plan is impacted and the patient may undergo palliative chemotherapy alone.

The previous examples illustrate that patients with the same FIGO stage may end up having different treatments based on the results of imaging studies. This makes comparing outcomes of such patients with the same disease stage impractical or impossible if the FIGO stage is the governing category. Thus, it is imperative that the staging system allows clinicians and outcomes researchers to distinguish patients who are fully staged using advanced imaging from those who are staged based on clinical examination alone. We suggest that this could be achieved by adding additional indicators for image-based staging or by full conversion to a TNM system.

The adoption of TNM staging for cervical cancer has the capacity to improve multidisciplinary cancer care on a population level, by allowing improved outcomes assessment, and on an individual level, by improving communication within the treatment team. When staging formally incorporates the information provided by contemporary imaging, radiologists can communicate their results within that structured framework and even issue an image-based TNM assessment.^22^ The impact is improved clarity,^23^ higher satisfaction among ordering physicians,^24^ and more impact on patient care from these often expensive imaging studies. Image-based staging must of course be integrated with clinical examination, pathologic results, and other data, but this should occur within a staging framework to which all specialties may contribute.

The ASCO-defined global resource-tiered treatment recommendations are an important step in standardizing cervical cancer care. It should be noted that the ASCO guidelines encourage health care providers and health care system decision makers to base treatment recommendations on the highest stratum of resources available.^3^ To enable and promote universal outcomes assessment for patients with cervical cancer, we propose a staging modification to FIGO that uses a three-tiered system to adjust for the ASCO-defined global resource setting (Table 1). Indicators such as BI for basic imaging, which represents the current FIGO staging for cervical cancer, LI for limited imaging, and AI for advanced imaging are suggested. Such a staging modification can greatly enhance our ability to accurately compare patient outcomes across varying resource settings.

**Table 1:**
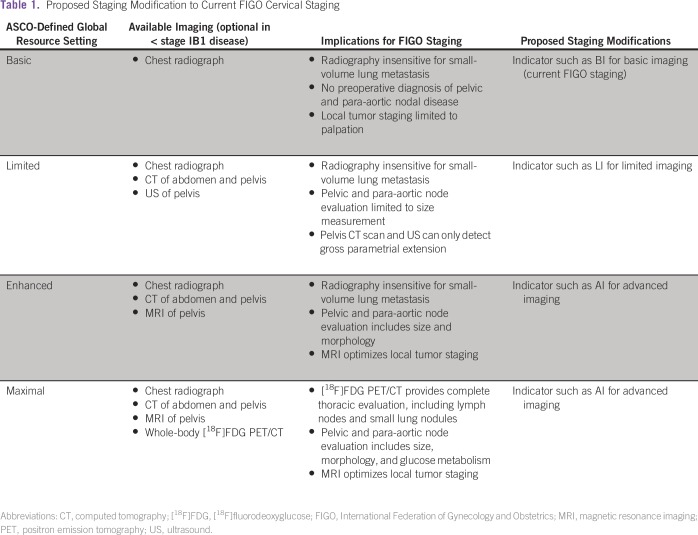
Proposed Staging Modification to Current FIGO Cervical Staging

## FIGO Staging and the Global Radiology Gap

Because cervical cancer is a bigger burden in developing nations with inconsistent availability and use of imaging, it is crucial to consider the ability of these regions to adapt to a modification of the staging framework. According to the data presented at the 2012 RAD-AID conference focused on international radiology for developing countries, quantifying the radiology gap remains difficult because of the complexity of measuring hardware, personnel, quality, and other components of radiologic services, but radiology shortages were estimated to affect 3.5 billion to 4.7 billion people.^25,26^

Yet, this major radiology gap exists for all patients with cancer. Other cancers predominating in less developed areas with limited imaging availability include gastric and hepatocellular carcinoma; nevertheless, both are staged using a TNM classification system worldwide. In fact, many centers in developing countries that are equipped to treat cancer with surgery or radiation also have access to some or all types of modern diagnostic imaging. If assessment of locoregional or distant disease is not possible, these patients are considered incompletely staged. In the TNM parameters, if nodal or distant metastatic disease is not assessed, an “x” distinction is applied, such as Nx or Mx.

In pursuit of improved women’s health, the responsibility of organizations like FIGO and others is to advocate for global technologic advancements in diagnosis and treatment. By highlighting the importance and value of advanced imaging techniques for women with cervical cancer, FIGO can further advocate for patients by encouraging developing nations to increase access to such imaging modalities. Further, guidelines need to be laid down to inform the use of appropriate imaging and prevent both overuse and misuse of sophisticated imaging technology. RAD-AID advocated economic development to build health care capacity in tandem with community economic progress and multidisciplinary educational strategies for broad-based radiology capacity advancement. Other strategies to consider include advancing technical solutions to leverage the use of wireless telecommunications and portable devices, including backpack ultrasound machines and CT, MRI, and PET units in mobile trailers, and improved dialogue between imagers, oncologists, and public health specialists for coordinating global health strategies.

In conclusion, to harness the power of population-level data, improved quality and availability of imaging technology, and electronic medical records to improve outcomes of women with cervical cancer, patients and their disease must be stratified by a staging system that reflects the complexity of the clinical data available. In concordance with the tiered levels of care concept, we do not suggest that the same imaging evaluation can be feasibly or affordably provided everywhere in the world, but instead that clinicians should be able to express staging information obtained anywhere, by whatever means are available, on an internationally applicable scale. Thus, we propose a tiered staging system to indicate the level of imaging resources available and used for staging. A robust staging system that incorporates the various levels of care being provided globally is crucial to outcomes evaluation and the subsequent success of treatment planning guidelines. FIGO staging for cervical cancer should be modified to allow for a more thorough evaluation of tumor spread using ultrasound, CT, MRI, and [^18^F]FDG PET/CT as deemed appropriate. The staging system should advance with the established imaging data that exist for the utility of MRI and [^18^F]FDG PET/CT, in particular for cervical cancer. Rather than lowering the ceiling of care by artificially constricting the information that contributes to accurate disease staging, we should raise the floor. To improve access to these advanced imaging modalities and utilization by gynecologic oncologists where the technology is available, FIGO and other organizations must continue strategies to improve health care capacity and endorse the contribution of technology by formally incorporating advanced imaging into the staging system.
